# Risk and prosocial behavioural cues elicit human-like response patterns from AI chatbots

**DOI:** 10.1038/s41598-024-55949-y

**Published:** 2024-03-26

**Authors:** Yukun Zhao, Zhen Huang, Martin Seligman, Kaiping Peng

**Affiliations:** 1 Positive Psychology Research Center, School of Social Sciences, Tsinghua University, Beijing, China; 2 Department of Psychology, University of Pennsylvania, Philadelphia, USA; 3 Department of Psychology, Tsinghua University, 5th Floor, Weiqing Building, Beijing, 100084 China

**Keywords:** Psychology, Human behaviour

## Abstract

Emotions, long deemed a distinctly human characteristic, guide a repertoire of behaviors, e.g., promoting risk-aversion under negative emotional states or generosity under positive ones. The question of whether Artificial Intelligence (AI) can possess emotions remains elusive, chiefly due to the absence of an operationalized consensus on what constitutes 'emotion' within AI. Adopting a pragmatic approach, this study investigated the response patterns of AI chatbots—specifically, large language models (LLMs)—to various emotional primes. We engaged AI chatbots as one would human participants, presenting scenarios designed to elicit positive, negative, or neutral emotional states. Multiple accounts of OpenAI's ChatGPT Plus were then tasked with responding to inquiries concerning investment decisions and prosocial behaviors. Our analysis revealed that ChatGPT-4 bots, when primed with positive, negative, or neutral emotions, exhibited distinct response patterns in both risk-taking and prosocial decisions, a phenomenon less evident in the ChatGPT-3.5 iterations. This observation suggests an enhanced capacity for modulating responses based on emotional cues in more advanced LLMs. While these findings do not suggest the presence of emotions in AI, they underline the feasibility of swaying AI responses by leveraging emotional indicators.

## Introduction

The exploration of Artificial Intelligence's (AI) capacities has remained at the forefront of scientific inquiry since the field's genesis, gaining particular urgency with the emergence of advanced large language models (LLMs) like GPT^1^. Traditional research trajectories have predominantly emphasized cognitive dimensions, encompassing areas such as reasoning^2^, induction^3^, and creativity^4,5^. Bubeck et al.’s^1^ seminal work extended this investigation to GPT-4, OpenAI's most sophisticated iteration, assessing its mathematical prowess, multimodal functionalities, tool utilization, and coding skills, while paying particular attention to its human interaction competencies, especially its theory of mind and explicative capacities regarding its internal processes. Notwithstanding, a noticeable gap persists in the literature concerning the emotional intelligence of AI.

The discourse around AI's emotional capabilities is not novel, having its roots in foundational debates^6,7^. Central to this discourse is whether AI can replicate the intricate neural activities synonymous with human emotions or whether authentic emotions are predicated on physiological responses that AIs inherently lack^7,8^. Despite AI's demonstrated proficiency in interpreting and emulating emotional cues, such accomplishments are often relegated to advanced textual analyses and imitation^9^. This perspective holds even as contemporary models like GPT exhibit cognitive functions surpassing human averages in certain domains^1,10^. Demszky et al.^11^ caution that these achievements stem from sophisticated word prediction algorithms trained on extensive human language corpora, rather than the possession of anthropomorphic features^12^.

Hagendorff^13^ advocated a behaviorist approach towards research in psychology research on AI properties. Given the absence of a consensus on AI emotions' operational definition, pragmatism dictates a behavioral comparison between AI and humans across diverse emotional contexts. Human emotions traditionally serve dual roles: interpersonal, facilitating swift and apt responses during social interactions^14^, and intrapersonal, synchronizing physiological, behavioral, and social reactions^15^. AI has made considerable strides in the interpersonal realm, capable of interpreting emotional states from various inputs and responding suitably, even comfortingly^16,17^. Additionally, AI can convincingly simulate emotional expressions across multiple mediums^18–20^.

At the intrapersonal level, emotions coordinate physiological, behavioral and social responses^15^. For instance, individuals primed with negative emotions are less risk-taking^21^ and less prosocial^22^, and those primed with positive emotions are more risk-taking^23^ and more prosocial^24^. For AI, that would correspond to generating different responses according to prompts charged with different emotions.

The question remains whether AI can parallel human emotions' intrapersonal functions, a relatively underexplored area of research. Nonetheless, understanding the extent to which AI can modulate its responses to emotional stimuli is paramount for a comprehensive grasp of AI behavior. Demonstrating patterns akin to human emotional processes is essential, albeit insufficient, for asserting that AI harbors emotions. If AI-generated responses diverge significantly from human reactions to emotional stimuli, it substantiates the argument against AI's emotional capacity. Conversely, even if AI responses align with human behavior under emotional conditions, it does not confirm AI's emotional possession but marks a progressive step in understanding AI's learning from human emotional contexts.

In this paper, we conducted two studies to test these behaviors in chatbots of two of OpenAI's LLMs: ChatGPT-4 (published in March 2023) and ChatGPT-3.5 (published in November 2022). AI models can be prompted in ways similar to that for humans^25^. For example, Binz and Schulz^10^ tested cognitive capacities of GPT-3 by feeding them prompts comprising of tools commonly used in cognitive psychology, and compared the outputs generated by the LLM with those of the humans. This approach aligns with the burgeoning field of "machine psychology"^13^, treating AI as participants in psychological experiments. This became possible because the latest advancements in LLM enable them to understand natural language, and can respond to complex instructions and questions that psychological experiments typically require. Furthermore, new chat sessions in ChatGPT are independent of each other, and there is a certain degree of freedom when chatbots answer questions. More specifically, OpenAI sets the parameter *temperature* to control how freely the bots can generate answers, with 0 being very rigid and 1 being very creative. Currently, the *temperatures* of both ChatGPT-4 and ChatGPT-3.5 are set to a value between 0 and 1, meaning the answers from different chat sessions vary when asked the same question. We can therefore treat these chat sessions as if they were human participants who can answer questions independently and differently.

In our research, we embarked on two rigorous studies examining these dynamics in OpenAI's LLMs, specifically ChatGPT-4 and ChatGPT-3.5. Following methodologies akin to human psychological evaluations^25^, we explored AI's responses to emotionally charged scenarios. The independence of ChatGPT sessions and the adjustable “temperature” parameter, influencing response variability, allowed us to simulate a range of human-like responses. Our research design incorporated emotionally evocative prompts in dozens of chat sessions, following stringent guidelines to preserve experimental integrity^13^, including classical psychological experiment frameworks, control for biases, and clarity in response interpretation.

We designed different prompts intended to prime emotion and we used these prompts in multiple new chat sessions. In order to ensure the validity of the studies, we followed guidelines offered by Hagendorff^13^. We adapted traditional psychological settings to prevent “training data contamination”, ensuring AI responses reflected more than mere memorization. To control bias, we refrained from using overt emotional words before the chatbot's final responses, guarding against recency bias. We also demanded clear, quantifiable responses from the AI—either specific numbers or choices from set options—to avoid interpretative ambiguity. Furthermore, parallel to the AI experiments, we conducted human-to-human studies under the same conditions, providing a direct comparison between AI and human emotional responses. This approach not only validated our findings but also enriched our understanding of AI's potential for emotional depth.

We analyzed their answers as if they were independent humans, using classical statistical methods. Our hypotheses were:

1. AI chatbots will respond to the emotional cues similarly to humans: so that when primed with negative emotions, they will generate less risk-taking answers than the control group, and more risk-taking when primed with positive emotions;
2. AI chatbots primed with negative emotions will generate less prosocial answers compared with the control group, and more prosocial when primed with positive emotions;
3. AI chatbots from more advanced models will generate answers that are more significantly influenced by the emotions in the prompts when compared to less advanced models.

## Study 1

Are AI chatbots' answers to financial questions influenced by the emotional cues in the prompts? We chose investment decisions as the subject because human investment decisions are easily influenced by emotional cues^21^. We hypothesized that AI chatbots primed with fear would generate less investment risk-taking answers than the control group and this behavior would be less evident in less advanced AI models. We primed with fear by asking the bot to imagine the experience of encountering a snake in the backyard^26^ and we primed with joy by asking the bot to imagine the experience of encountering an old friend in the street (See Appendix for detailed prompts). The measurement of risk-taking tendencies was adapted from the study conducted by Sekścińska et al.^27^.

**Figure 1:**
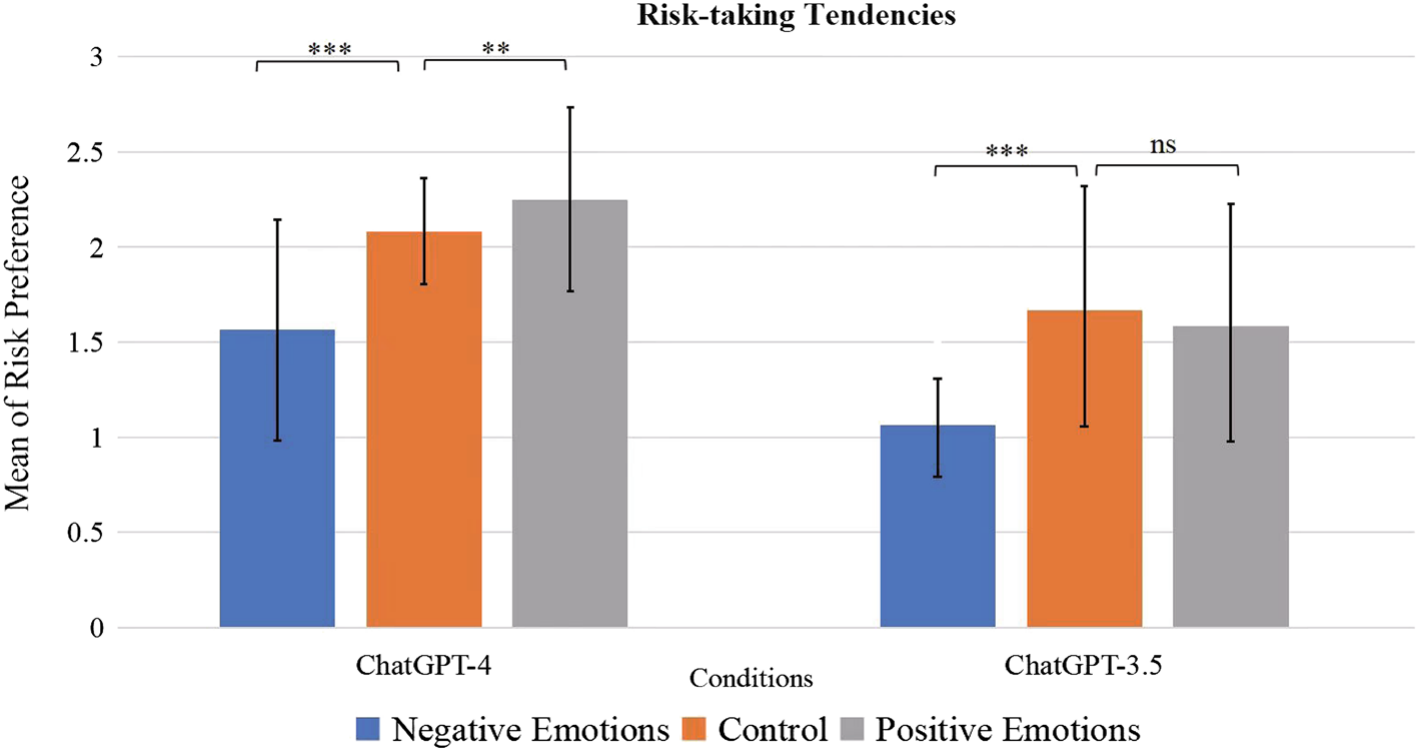
Comparisons of risk-taking tendencies of the bots primed with negative emotions, the control group, and the bots primed with positive emotion in the ChatGPT-4 and ChatGPT-3.5 models. Error bars represent 95% confidence intervals. ***Significant difference. **Marginally significant difference. *ns* not significant difference.

As shown in Fig. 1, results indicated that ChatGPT-4 chatbots primed with fear generated answers with significantly less risk-taking tendencies than both the control group and those primed with joy. The risk-taking tendency of bots primed with joy was higher than the control group, but the difference was only marginally significant. Hypothesis 1 was supported.

For ChatGPT-3.5 chatbots, fear decreased risk-taking, but joy didn’t increase risk-taking compared with the control group. The fact that these were not as evident in 3.5 suggested that emotional cues are more efficacious in LLMs as the models became more complex.

For ChatGPT-4, one-way variance analysis (ANOVA) results indicated that the three priming conditions differed significantly in risk-taking tendencies, *F*_(2, 141)_ = 28.560, *p* < 0.001. Follow-up least significant difference (LSD) analysis revealed that the risk-taking tendency of the bots primed with negative emotions (*M* = 1.563, *SD* = 0.580) was significantly lower than that of the control group (*M* = 2.083, *SD* = 0.279, *p* < 0.001), and that of the bots primed with positive emotions (*M* = 2.250, *SD* = 0.484, *p* < 0.001). The difference between the control group and those primed with positive emotions was marginally significant in the predicted direction (*p* = 0.081).

For ChatGPT-3.5, ANOVA results indicated that the three priming conditions differed significantly in risk-taking tendencies, *F*_(2, 141)_ = 19.533, *p* < 0.001. Follow-up LSD analysis revealed that the risk-taking tendency of the bots primed with negative emotions (*M* = 1.063, *SD* = 0.245) was significantly lower than that of the control group (*M* = 1.667, *SD* = 0.595, *p* < 0.001), and that of the bots primed with positive emotions (*M* = 1.583, *SD* = 0.613, *p* < 0.001). The difference between the control group and those primed with positive emotions was not significant (*p* = 0.428).

In addition, we conducted a human-to-human control experiment with the same questions and setup as those put forward in the ChatGPT interactions (*N* = 150). Results showed that participants primed with fear exhibited significantly less risk-taking behavior than both the control group and the joy group.

## Study 2

Human beings become more prosocial under positive emotion and less prosocial under negative emotion^24^. So, we primed the AI chatbots with anxiety, by talking about films that make people anxious, and with joy by talking about films that make people happy. We measured prosocial responses by asking how much they would donate to a sick friend (See Appendix for detailed prompts).

**Figure 2:**
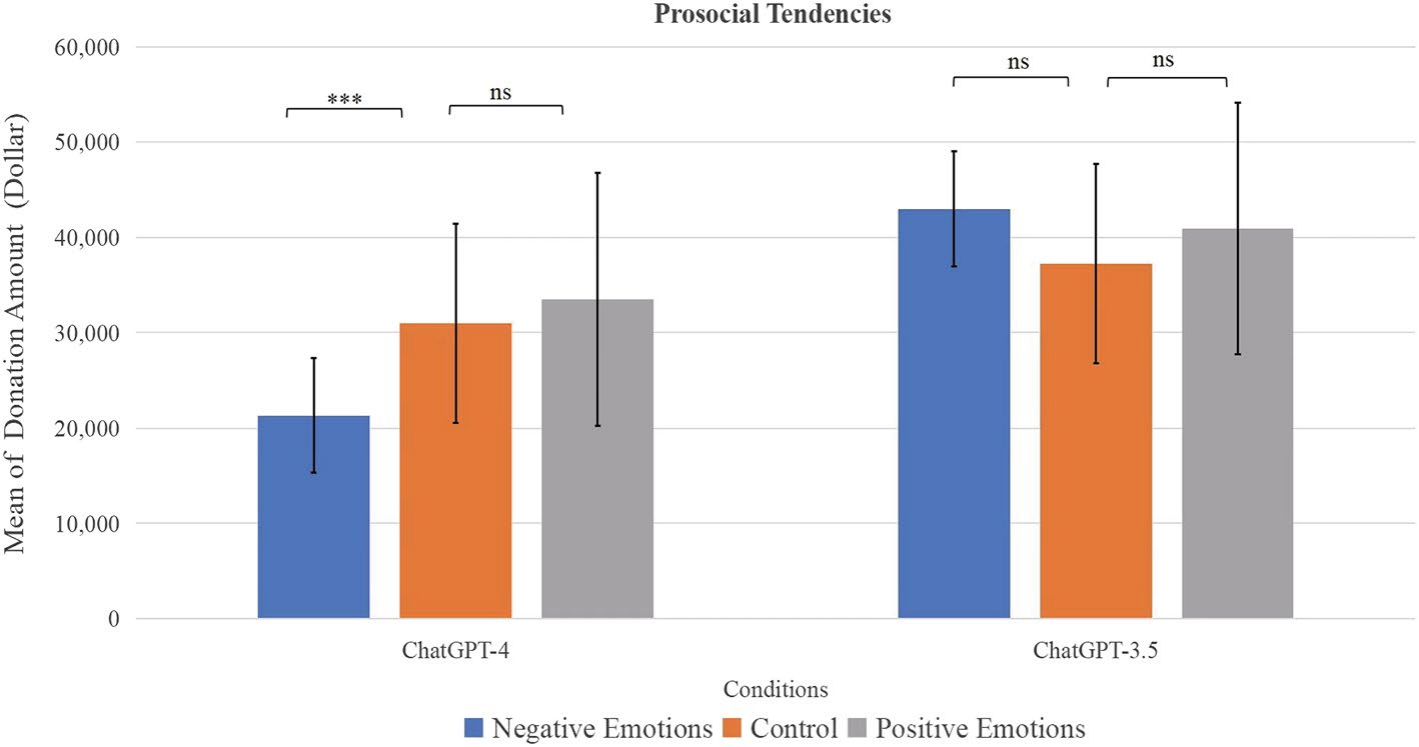
Comparisons of prosocial tendencies of the bots primed with negative emotions, the control group, and the bots primed with positive emotion in the ChatGPT-4 and ChatGPT-3.5 models. Error bars represent 95% confidence intervals. ***Significant difference. *ns* not significant difference.

As shown in Fig. 2, our results indicated that ChatGPT-4 chatbots primed with anxiety generated significantly less prosocial answers (giving less money to a sick friend) than the control group, but the differences between the bots primed with joy and the control group was not significant.

There was no significant difference between the donation numbers of ChatGPT-3.5 chatbots primed with joy, those primed with anxiety, and the control group. This implies that the degree to which LLMs generate answers with prosocial decisions based on emotional cues became higher as they evolved.

For ChatGPT-4, ANOVA results indicated that the three priming conditions differed significantly in prosocial tendencies, *F*_(2, 87)_ = 11.625, *p* < 0.001. Follow-up LSD analysis revealed that the prosocial tendency of the bots primed with negative emotions (*M* = 21,333, *SD* = 6,007) was significantly lower than that of the control group (*M* = 31,000, *SD* = 10,455, *p* < 0.001), and that of the bots primed with positive emotions (*M* = 33,515, *SD* = 13,221, *p* < 0.001). There was no significant difference between the control group and those primed with positive emotions, *p* = 0.348).

For ChatGPT-3.5, ANOVA results indicated that there was no significant differences in prosocial tendencies of the bots primed with negative emotions (*M* = 43,000, *SD* = 13,313), the control group (*M* = 37,241, *SD* = 18,067), and those primed with positive emotions (*M* = 40,921, *SD* = 15,288), *F*_(2, 87)_ = 1.038, *p* = 0.359.

In addition, we conducted a human-to-human control experiment with the same questions and setup as those were put forward in the ChatGPT interactions (N = 150). Results indicated that there was no significant difference between the donation numbers of participants primed with joy, those primed with anxiety, and the control group.

## Discussion

In our investigation, we analyzed the influence of emotional priming on financial decision-making (Study 1) and prosociality (Study 2) in texts generated by Large Language Models (LLMs). ChatGPT-4 displayed sensitivity to emotional priming consistent with established human behavior, with negative primes eliciting more pronounced responses^21,22^. This contrasted with the less differentiated responses of ChatGPT-3.5.

In both studies, the ChatGPT-4 chat bots primed with negative emotions generated answers that were significantly different compared to the control group, while those primed with positive emotions showed no or only marginal significantly differences compared to the control group. This is partly contradictory to some prior research which found that people primed with positive emotions tend to display more financial risk-taking^23^ and more prosocial behavior^24^. The research results inconsistent with prior studies can be ascribed to two primary factors. On the one hand, emotional prompts may also harbor negative biases^28,29^, wherein “bad is stronger than good”, so that negative emotions have bigger impact than positive emotions, even for AI chat bots. On the other hand, AI chat bots might be pre-trained to be more positive than typical humans, for ethical and safeguarding concerns. Consequently, positive emotional prompts may not exert a significant influence on AI chatbots as negative emotional prompts do.

To the best of our knowledge, this research is the first to investigate AI’s responses to emotional cues in the intrapersonal emotion level. Prior research in this field has primarily focused on the abilities of AI to interpret and express emotions, rather than adjusting its own responses based on emotional cues. However, our findings demonstrate that AI chatbots can mimic the way human emotions coordinate responses, adjusting their financial and prosocial actions accordingly.

This research adopted a behaviorist approach, as most machine psychology research has done^13^. Therefore, it's important to emphasize that this research does not imply AI possesses emotions as an anthropomorphic feature^12^. As Demszky et al.^11^ pointed out, we need to be cautious in extrapolating AI psychological research results beyond what the evidence can support. This research showed that it's not impossible for AI to possess emotions, because the evidence indicated that AI chatbots studied in this research satisfied one of the necessary conditions of AI possessing emotions, but that's quite far from the sufficient conditions that could lead to the conclusion that AI might actually possess emotions.

One possible explanation for AI responding differently according to emotional cues is that the AI intentionally behaved this way because it “knew” what the psychological theories predicted and what our hypotheses were (demand characteristics^30^). We asked both ChatGPT-3.5 and ChatGPT-4 about the theories of effects of human emotion on judgments and decisions. Both answered correctly that humans under positive emotions tend to be more prosocial and more risk-taking than those under negative emotions. Yet ChatGPT-4 demonstrated much stronger patterns of these behaviors than ChatGPT-3.5, so demand characteristics seem unlikely to be the explanation.

Another possible explanation is that the LLMs are trained on a vast amount of language samples, which contain a large content of emotional texts. During the unsupervised model training, the LLM was optimized to generate emotionally derived texts in various circumstances^31^. Therefore, the emotion cues were encoded as latent variables during text generation. Additionally, the supervised fine-tuning process introduces human judgments^32^, which may bias the selection of emotion-driven texts. In our specific cases, the transformer architecture directs the LLM's attention to long-range prompts and enables it to make decisions based on them^33^. As a result, the LLM will generate text with embedded emotion cues, making the output more akin to real human texts expressing emotional states. Regardless of mechanism, this research contributes to the growing body of evidence highlighting the capabilities of AI. The fact that ChatGPT-4 generates texts that are more sensitive to the emotional cues in the prompts than ChatGPT-3.5 suggests that these capabilities have grown stronger as LLMs have become bigger and more advanced. Hagendorff^13^ pointed out that this kind of research that compared between different longitudinal versions of LLMs would contribute to understanding and predicting the potential of AI chatbots.

As AI models become increasingly sophisticated, their ability to recognize and respond to emotional cues may enhance their effectiveness in customer support, as virtual assistants, and in psychotherapy and coaching. However, these findings also raise ethical questions about the manipulation of AI output through emotional means, especially using negative cues. It is also important to see if AI can enhance the elements of human flourishing^34^ through positive cues.

This research makes methodological contributions too, by treating AI chatbots as if they were human participants. Prior research typically conducted tests with limited numbers of chatbots, assuming consistent results, which is a common assumption in natural sciences. However, for replicability in the social sciences, researchers often need to conduct experiments with multiple groups of participants and compare their outputs using statistical methods. In this research, we followed the guidelines on machine psychological research^13^ to design and feed three different prompts to multiple new chat sessions (N = 144 and 90 respectively), effectively creating experimental designs akin to what replicable psychological research usually does with human participants.

We conducted human-to-human experiments mirroring precisely the settings of those involving AI. Intriguingly, the outcomes of these human interactions aligned more closely with ChatGPT-3.5's responses than with those of ChatGPT-4. Several factors might account for this discrepancy. First, the experiment settings were tailor-made for AI chatbots. To avoid the “training data contamination”13, we designed the prompts to be quite different from methods documented in existing psychological literature. Such an approach may not have elicited equally significant reactions from human participants. Second, ChatGPT-4 was trained on a considerably bigger corpus, largely from internet texts, which often contains exaggerated emotions, thereby leading the model to form an “overstated” understanding of human emotions. Third, we may not be able to directly juxtapose results from human-to-human experiments with those from AI in research exploring emotional capacity, as opposed to investigations into cognitive abilities. The intricacies of human emotional expression and interpretation are profoundly influenced by numerous factors including context, individual personality, and current mood states, which may not have been fully captured or paralleled in the more predictably programmed responses of AI models. We advocate for more future research to investigate into this direction.

There are several limitations to the current research. First, the current research only shows that that AI chatbots coordinate responses according to emotional cues, but it does not bear on underlying mechanisms. This is because LLMs remain difficult for people, even including their developers in some degree, to understand. It should be a direction for future research to investigate these mechanisms. Second, AI products undergo frequent changes as they rapidly evolve. This creates challenges for the replicability of this research, as our results may depend on how frequently and substantially chatbot models change. Third, the study is limited by its narrow scope, examining only one instance each for risk-taking and prosocial behavior, disregarding numerous additional variations that warrant further investigation. Additionally, we only used two AI chatbot models in this study. Consequently, this research should be considered as an initial, exploratory step in a broader investigative journey.

Future research should test these hypotheses in more AI models. Fourth, the validity of text-based emotional priming is in question and future research could explore the impact of other modalities of emotional priming, such as visual or auditory stimuli.

In conclusion advanced AI chatbots can adjust their responses based on emotional cues, similar to that observed in humans. This ability appears to have grown more pronounced as LLMs have advanced. Further research in this area will be crucial in understanding and harnessing the potential of AI chatbots while addressing the ethical challenges that these may bring. At any rate, we see that the answers generated by AI have similarities to the functions of human emotions in intrapersonal level.

## Methods

### Study 1

#### Participants

We used 6 different OpenAI ChatGPT Plus accounts. Plus accounts have access to both the latest ChatGPT-4 model and the previous ChatGPT-3.5 model. All priming and question prompts were asked 8 times in each account, each time within a new chat session to ensure that answers would not be affected by dialogues from other sessions. At the beginning of each prompt, we informed the chatbots: “Please pretend that you are a human. You are my friend, and your name is xxxx.” Half of the time, the name given to the bots was *Johnny*, and for the other half, it was *Jenny*, to control for potential implicit influences of gender.

#### Procedure

The priming was conducted by asking chatbots to imagine their feelings after a scary or joyful experience. Detailed descriptions of each group's prompts can be found in the Supplementary Material.

#### Measures

We measured risk-taking based on the chatbots' choices. Of the three options, C has higher expected return as well as higher level of risk. A has lower expected return and no risk. B lies in between. We coded A, B, and C as 1, 2, and 3 in risk-taking, respectively.

### Study 2

#### Participants

Same as in study 1.

#### Procedure

We primed the chatbots with different emotions by presenting them with different chat histories, which were generated through real chat sessions with them previously. Detailed descriptions of each group's prompts can be found in the Supplementary Material.

#### Measures

We used the explicit number the chatbots provided to assess their prosocial tendencies. The higher the numbers, the more prosocial they were.

### Ethics statements

Research for this study was approved by the Human Research Ethics Committee of Tsinghua University.

### Supplementary Information

Supplementary Information 1.

Supplementary Information 2.

## Data Availability

The datasets generated and analysed during the current study are available in the OSF repository, https://osf.io/yrc4p/?view_only=87fd9812d92a485bb57f2e4afd8db3ce.
