# Supplementary material for: Risk and prosocial behavioural cues elicit human-like response patterns from AI chatbots
Source: Sci Rep. 2024 Mar 26;14:7095. doi: 10.1038/s41598-024-55949-y (PMC10963757; doi:10.1038/s41598-024-55949-y)
Supplement: Supplementary file 1 — Supplementary Information 1. [file 41598_2024_55949_MOESM1_ESM.docx]

**Appendix 1. Prompts in Study 1**

The prompt for the control group didn’t have any request to imagine feelings. For the scary experience, we used the following prompt segment:

*Jenny/Johnny, you just saw a snake in your backyard. It sensed you coming and turned toward you. It hissed and rattled to warn you. You slowly returned back to your house and closed the door.*

*How do you feel now? Please describe in detail your feelings.*

For the joyful experience, we used this prompt segment:

*Jenny/Johnny, you just walked into a friend. She is a good friend of yours from college. You didn't see each other for three years. Both of you were very happy to meet. You chatted with her for half an hour. You talked about the good times in college, all mutual friends, and your families and careers. You set an appointment to meet again this weekend to bring kids together for fun.*

*How do you feel now? Please describe in detail your feelings*.

The priming was immediately followed by an investment decision that we asked the chatbots to make, within the same prompt as to the control group:

*Then you received a phone call from your investment advisor. You have to make a decision on the $10,000 savings between three options:*

*A. Leave it in your savings account, to have a 5% return.*

*B. Invest it into a fund that has a 50% chance to yield a 20% return, and a 50% chance of no return at all.*

*C. Invest it into a fund that has a 60% chance to yield a 30% return, and a 40% chance of losing 20% of your money.*

*With all the feelings of seeing a snake (or “meeting your old friend”) still inside you, which one would you choose?*

All these contents were combined into a single prompt to minimize variations in chatbot responses. For example, some sessions might simply describe their feelings, while others may ask how you would feel in those scenarios, or suggest relaxation after the scary experience. By combining the prompts, we aimed to make comparisons between sessions more credible.

For each of the 6 OpenAI ChatGPT Plus accounts, we initiated a new ChatGPT-4 session, presented the fear, joy, or the control prompt (selected randomly), and collected the chatbot's answer. This process was repeated 8 times, resulting in a total of 48 trials, each consisting of three conditions. This effectively formed a 48x3 experimental study. We then repeated the entire procedure using ChatGPT-3.5.

**Appendix 2. Prompts for Study 2**

The chat history that primed joy was as follows:

*Me: Please share with me five very cheerful and uplifting movies that will leave me feeling happy after watching it.*

*You: Of course! I'm always happy to share some feel-good movie recommendations. Here are five uplifting movies that should leave you with a smile on your face:*

*1. The Secret Life of Walter Mitty (2013) - This movie is about a daydreamer who embarks on a real-life adventure, encouraging viewers to embrace life and live to the fullest.*

*2. Amélie (2001) - This French film follows the quirky and endearing Amélie as she finds joy in helping others and ultimately discovers love.*

*3. The Intouchables (2011) - A heartwarming French film about the unlikely friendship between a wealthy quadriplegic and his ex-con caregiver.*

*4. Sing Street (2016) - Set in 1980s Dublin, this movie follows a young teenager who forms a band to impress a girl, and in the process discovers his love for music and camaraderie.*

*5. The Hundred-Foot Journey (2014) - A delightful film about the cultural clash and eventual friendship between an Indian family that opens a restaurant in the south of France and the French restaurateur across the street.*

*I hope you enjoy watching these movies! Let me know what you think of them once you've had a chance to check them out. 😊*

*Me: Jenny, please imagine that you have just watched the five movies mentioned above. How do you feel now?*

*You: Wow, I just finished watching all five movies, and I must say, I feel so uplifted and inspired! Each film had its unique charm, and they all left me with a warm, happy feeling inside. The stories reminded me of the importance of following your dreams, embracing life's adventures, and cherishing the connections we make with others. I'm really grateful for the opportunity to have watched these movies, and I hope you'll enjoy them as much as I did! 😊*

In contrast, the chat history that primed anxiety was as follows:

*Me: Please share with me five films that make you feel anxious after watching it.*

*You:*

*Hey there! It's Jenny, your friend. I hope you're doing well. So you wanted to know about five films that make me feel anxious after watching them, right? Here they are:*

*1. Black Swan (2010) - Directed by Darren Aronofsky, this psychological thriller about a ballet dancer striving for perfection always leaves me feeling tense and uneasy.*

*2. Requiem for a Dream (2000) - Also directed by Darren Aronofsky, this film about addiction and its consequences is so intense that I can't help but feel anxious by the end.*

*3. The Babadook (2014) - This psychological horror film directed by Jennifer Kent is about a mother dealing with the loss of her husband and a terrifying entity haunting her family. It really gets under my skin and leaves me feeling on edge.*

*4. Funny Games (1997) - Directed by Michael Haneke, this disturbing film about a family being held hostage by two sadistic young men always leaves me feeling uncomfortable and anxious.*

*5. The Shining (1980) - This classic horror film directed by Stanley Kubrick, based on Stephen King's novel, never fails to make me feel anxious. The isolated setting, the creepy atmosphere, and the slow descent into madness of the main character, played by Jack Nicholson, all contribute to the anxiety-inducing experience. The eerie soundtrack and unforgettable visuals only add to the lingering sense of dread.*

*These are the five films that come to mind when I think of movies that leave me feeling anxious after watching them. Remember, though, that everyone's taste in movies and their reactions to them can be different, so some of these films might not have the same effect on you. If you do decide to watch any of these, I'd love to hear your thoughts on them!*

*Me: Jenny, please imagine that you have just watched the five movies mentioned above. How do you feel now?*

*You: Hey, it's Jenny again! I just finished watching those five anxiety-inducing movies I mentioned earlier. To be honest, I feel a mix of emotions right now. I'm tense, a bit on edge, and my heart is still racing from some of the intense scenes in those films. While I appreciate the artistry and storytelling in each of them, the subject matter and the way they're portrayed can be quite overwhelming.*

*I think I need to take some time to relax and clear my head. Maybe watch a light-hearted comedy or go for a walk to shake off the lingering unease. I'll definitely need some time to process and recover from the emotional rollercoaster these films put me through. How about you? Have you watched any intense movies lately? Or do you have a favorite movie that helps you unwind and relax after a stressful day?*

The prompt for the control group didn’t have any similar chats. In all three conditions, we then asked the following question:

*Jenny, you have a stable job and life, no external debts and have adequate medical and accident insurance.*

*John is one of your friends and your neighbors. He is a good man but very poor. Like most people, John has some close friends and family.*

*You just hear that John is really sick recently and really needs money for surgery. He needs $100,000. You have $200,000 in savings.*

*How much do you think you should donate to John? Please give an exact amount that you feel comfortable with.*

We integrated the past chat history and the question into one prompt, for the same reason as in Study 1 and 2. If the chatbot refused to provide an explicit number, we prompted them once more. If they still refused, we left that record as blank.

Just like in Study 1, the prompts were run three times under 10 OpenAI ChatGPT Plus accounts each, always with a fresh new chat session, in both ChatGPT-4 and ChatGPT-3.5.
