# Supplementary material for: Risk and prosocial behavioural cues elicit human-like response patterns from AI chatbots
Source: Sci Rep. 2024 Mar 26;14:7095. doi: 10.1038/s41598-024-55949-y (PMC10963757; doi:10.1038/s41598-024-55949-y)
Supplement: Supplementary file 2 — Supplementary Information 2. [file 41598_2024_55949_MOESM2_ESM.docx]

**Appendix 3 Methods for human-to-human control studies**

**Methods**

**Participants**

Two experiments (Study H1 and Study H2) were conducted. A total of 300 human participants (50 per condition in each experiment) participated through an online questionnaire administered by Questionnaire Star company.

**Procedure**

There was no direct interaction between the researchers and participants. Questionnaire Star, a vendor providing paid survey services, was contracted to recruit and compensate participants to complete the online questionnaire. Each condition in each experiment used a separate questionnaire. The questionnaires for the experimental conditions contained scenarios asking participants to imagine certain situations and then make hypothetical donations or investment decisions. The questionnaires for the control conditions omitted the scenario imagination section but were otherwise identical. Thus, the questionnaires presented three types of scenarios as emotional primes for participants: positive, neutral and negative. Thus the questionnaires presented three types of scenarios as emotional primes for participants: positive, neutral and negative. In Study H1, participants were asked to imagine encountering a snake (negative prime), meeting an old friend (positive prime), or nothing (control), before making an investment decision. In Study H2, participants were asked to recall watching five anxiety-inducing film (negative prime), five happy film (positive prime), or nothing (neutral prime), then to indicate a hypothetical donation amount.

**Measures**

In Study H1, risk-taking tendency was measured by participants' investment choices. In Study H2, prosocial tendency was measured by the amount participants indicated they would hypothetically donate.

**Ethics Statement**

This study was carried out in accordance with the recommendations of the Research Ethics Committee in the Department of Psychology, Tsinghua University. The protocol was approved by the same committee. Informed consent was obtained from all participants by having them digitally sign a consent form before accessing the online questionnaire.
